# Pleiotropic effects of a recessive *Col1a2* mutation occurring in a mouse model of severe osteogenesis imperfecta

**DOI:** 10.1371/journal.pone.0309801

**Published:** 2025-02-05

**Authors:** Michelangelo Corcelli, Rachel Sagar, Ellen Petzendorfer, Mohammad Mehedi Hasan, Fleur S. van Dijk, Anna L. David, Pascale V. Guillot

**Affiliations:** 1 Research Department of Maternal and Fetal Medicine, Elizabeth Garrett Anderson Institute for Women’s Health, University College London, London, United Kingdom; 2 Northwest Thames Regional Genetics Service, London Northwest University Healthcare NHS Trust, London, United Kingdom; 3 Department of Metabolism, Digestion and Reproduction, Section of Genomics and Genetics, Imperial College London, London, United Kingdom; 4 Department of Development and Regeneration, Katholieke Universiteit Leuven, Leuven, Belgium; University of Vermont College of Medicine, UNITED STATES OF AMERICA

## Abstract

In Europe, approximately 85–90% of individuals with Osteogenesis Imperfecta (OI) have dominant pathogenic variants in the *Col1a1 or Col1a2* genes whilst for Asian, especially Indian and Chinese cohorts, this ratio is much lower. This leads to decreased or abnormal Collagen type I production. Subsequently, bone formation is strongly reduced, causing bone fragility and liability to fractures throughout life. OI is clinically heterogeneous, with the severity ranging from mild to lethal depending on the gene and the type and location of the OI-causative variant and the subsequent effect on (pro) collagen type I synthesis. However, the specific effects on the phenotype and function of osteoblasts are not fully understood. To investigate this, one of the OI murine models was used, i.e. the *oim*/*oim* (OIM) mice, which closest resembling severely deforming OI in humans. We showed that in OIM, the *Col1a2* mutation results in a multifactorial inhibition of the osteogenic differentiation and maturation as well as inhibition of osteoclastogenesis. The phenotype of differentiated OIM osteoblasts also differs from that of wild type mature osteoblasts, with upregulated oxidative cell stress and autophagy pathways. The extracellular accumulation of defective type I collagen fibres contributes to activation of the TGF-β signalling pathway and activates the inflammatory pathway. These effects combine to destabilise the balance of bone turnover, increasing bone fragility. Together, these findings identify the complex mechanisms underlying OI bone fragility in the OIM model of severe OI and can potentially enable identification of clinically relevant endpoints to assess the efficacy of innovative pro-osteogenic treatment for patients with OI.

## Introduction

Bones provide structural support to the body, protection of vital organs, function as the main site of haematopoiesis, and play a key role in the regulation and storage/release of minerals, growth factors and hormones [1].

Osteogenesis imperfecta (OI), or brittle bone disease, is a rare monogenic inherited connective tissue disorder with a prevalence of 6-7/100,000 [2]. The main features of OI in addition to an increased risk of fractures throughout life include osteopenia, skeletal deformity, blue sclerae, hearing loss, dentinogenesis imperfecta, and short stature [3]. In Europe, approximately 85–90% of individuals with OI have a heterozygous pathogenic variant in the *Col1a1* or *Col1a2* genes whilst for Asian, especially Indian and Chinese cohorts, this ratio is much lower. These chains intertwine in a triple helix structure to form (pro)collagen type I. Depending on the gene(s) involved, the location and the type of variant, there is either a 50% decrease of (pro)collagen type I synthesis which leads to OI type 1 (haploinsufficiency) or there is production of abnormal (pro)collagen type I which usually leads to severe OI types. The most common types of variants causing *Col1a1*/*Col1a2* severe OI, are glycine substitutions as (pro)collagen type I has a large, conserved domain consisting of Gly-X-Y triplets. Glycine is the smallest amino acid and when substituted for a different amino acid this hampers proper folding and assembly of the triple helix [4, 5] which results in severe OI. Currently, more than 1000 different pathogenic variants have been reported in *Col1a1*/*Col1a2* [3].

In OIM (osteogenesis imperfecta murine) mice (*B6C3fe-a/a-oim)*, a recessive point mutation (a G deletion at nucleotide 3983 in *Col1a2*) in the gene coding for the alpha 2 chain of type I (pro)collagen (*Col1a2*) results in an alteration of the sequence of the last 48 amino acids, leading to the absence of Col1a2(I) polypeptide chain, despite mRNA transcription. In homozygous OIM (*oim/oim*), this leads to the formation of homotrimeric α1(I)_3_ type I collagen, instead of the normal heterotrimeric collagen α1(I)_2_α2(I)_1_. This recessive variant has the same effect in humans, namely structurally abnormal collagen type I being produced. The impairment of OIM osteoblastic differentiation and the increased osteoclast formation [6, 7] as well as the activation of the TGF-β signalling pathway [8] have previously been reported [9]. Using the α2(I)-G610C mouse model of OI, Mirigian et al. [10] also showed that osteoblast malfunction appeared to be caused by the cell stress response to procollagen misfolding. The presence of cell stress in OI has also been reported in other OI models for dominant and recessive OI. In dominant OI, the production of abnormal collagen chains often causes intracellular retention and aggregation, leading to ER stress. This stress activates the unfolded protein response (UPR), which is a cellular mechanism aimed at restoring normal ER function but can also lead to cell death if the stress is prolonged or severe. Research has shown that this ER stress is a significant factor in the pathogenesis of dominant OI, affecting cellular differentiation and bone form [11]. For recessive OI, which is caused by mutations in genes like *Crtap*, *Lepre1*, and *Ppib*, the proper folding and modification of collagen are disrupted. This leads to the accumulation of misfolded proteins in the ER, causing ER stress and activating the UPR. Studies have identified deficiencies in proteins such as OASIS (CREB3L1), which is an ER-stress transducer, as being responsible for severe forms of recessive OI [12]. The resulting ER stress can impair the maturation and function of osteoblasts [13].

To propose a theoretical model of OI bone fragility, we used primary pre-osteoblasts isolated from the calvaria of homozygous OIM and wild type (WT) neonatal mice to determine the genetic impact of the recessive variant on osteoblast phenotype during the process of osteogenic differentiation.

## Material and methods

### Animals

All experimental protocols complied with the UK Home Office guidelines (PPL 70/6857). Heterozygous male and female (B6C3Fe a/a-Col1a2oim/Col1a2oim) mice (Jackson Laboratory) were housed in individual ventilated cages at 21°C with a 12:12-hour light dark cycle. Offspring were genotyped by sequencing the OIM fragment then homozygous and wild type colonies established. For the analysis of the neonatal mice, the pups were culled at 7 days postnatal while adult animals were culled at 8-week postnatal.

### Primary osteoblast precursors isolation and expansion

Murine neonatal calvaria were harvested from 4–7 days old OIM and WT mice. The skulls were washed in PBS (Phosphate Buffered Saline) and transferred onto a sterile dish for dissection. A knife blade was used to scrape off any soft tissues. The bone tissue was then sequentially digested in a solution of 0.2% collagenase type II (Gibco) for a total of 1h 30min. The final digest was placed in a plastic cell culture dish with alpha modified Eagle’s medium (alpha-MEM) (Invitrogen) supplemented with 10% fetal bovine serum (Gibco), 2 mM L-glutamine, 50 IU/ml penicillin and 50 mg/ml streptomycin (Gibco), at 37°C in a 5% CO_2_ incubator. Cells were expanded for about a week until there was a homogeneous population of ALP (alkaline phosphatase) positive cells at sub-confluency. Cells were expanded and used immediately (p1) without further passaging, to prevent loss of osteogenic potential.

### *In vitro* osteogenic differentiation

Cells were differentiated down the osteoblast lineage for up to 4 weeks in alpha-MEM supplemented with 2 mM β-glycerophosphate, 0.2 mM ascorbic acid and 10^−8^ M dexamethasone, then fixed in 4% paraformaldehyde in PBS.

### Quantification of mineralised bone formation

Cells were stained with a solution of 1% Alizarin Red to visualize the calcium and phosphate deposits. Once fully dry, the plates were then scanned on a high-resolution flat-bed scanner (Epson Perfection v600). ImageJ free software was used to measure the area fraction (%) of the mineralised bone nodules.

### Micro-computed tomographic analysis

Tibiae were isolated from 1-week-old homozygous OIM mice (n = 7), 1-week-old wild type (WT) mice (n = 7), 8-week-old homozygous OIM mice (n  =  6) and 8-weeks old wild type (WT) mice (n  =  6). The bones were fixed in 10% neutral buffered formalin for 24 h, then washed in phosphate buffered saline and stored in 70% ethanol until scanning. All the scans were performed using a Skyscan 1172 μ-CT scanner (Bruker, Belgium). The bones were scanned at 50 kV and 200 μA using a 0.5 mm aluminium filter and a pixel resolution of 4.3 μm (for the 8-weeks old mice) and 3.3μm (for the 7-days old pups). To analyse the trabecular bone, a region of interest of the length of 0.8 mm was selected below the growth plate of the proximal tibia. To analyse cortical bone, a 0.4 mm long region of interest was selected with an offset of 2 mm below the growth plate. The images were reconstructed using the Skyscan NRecon software and the following cortical and trabecular morphometric parameters were calculated using the Skyscan CT Analyzer (CTAn) software: percent bone volume (BV/TV) (%), bone surface density (BS/TV), trabecular thickness (mm), trabecular number (mm^−1^), trabecular pattern factor (mm^−1^), intersection surface (mm^2^), cortical volume (mm^3^), medullary volume (mm^3^), cortical thickness (mm), total porosity (%), medullary canal thickness (mm), and tissue mineral density (TMD) (g/cm^3^). Bone mineral density was measured using 0.25 and 0.75 g/cm^3^ calcium hydroxyapatite calibration phantoms (Bruker) as a reference. P values were calculated using analysis of variance (one-way ANOVA) followed by Bonferroni’s multiple comparison post hoc test. Differences with a P-value of < 0.05 were considered significant. Data were expressed as mean ± SEM (standard error of the mean).

### Transcriptomic analysis (RNA-seq)

Total RNA was extracted from OIM and WT pre-osteoblasts and mature osteoblasts using TRIzol (Invitrogen). RNA sequencing (RNA-seq) analysis was performed (Genewiz), to investigate gene-expression profiling in the following groups (OIM pre-osteoblast, WT pre-osteoblast, OIM osteoblast, WT osteoblast). Differential gene expression analyses for all the group comparisons were performed using DESeq2 [14]. The Wald test was performed to obtain adjusted p-values and log2 fold changes. Genes with an adjusted p-value < 0.05 and absolute log2 fold change > 1 were considered differentially expressed genes for each comparison.

## Results

### Micro computed tomography (microCT) analysis of the proximal tibia of neonate and adult OIM mice shows deterioration of bone microarchitecture in older mice

**Fig 1:**
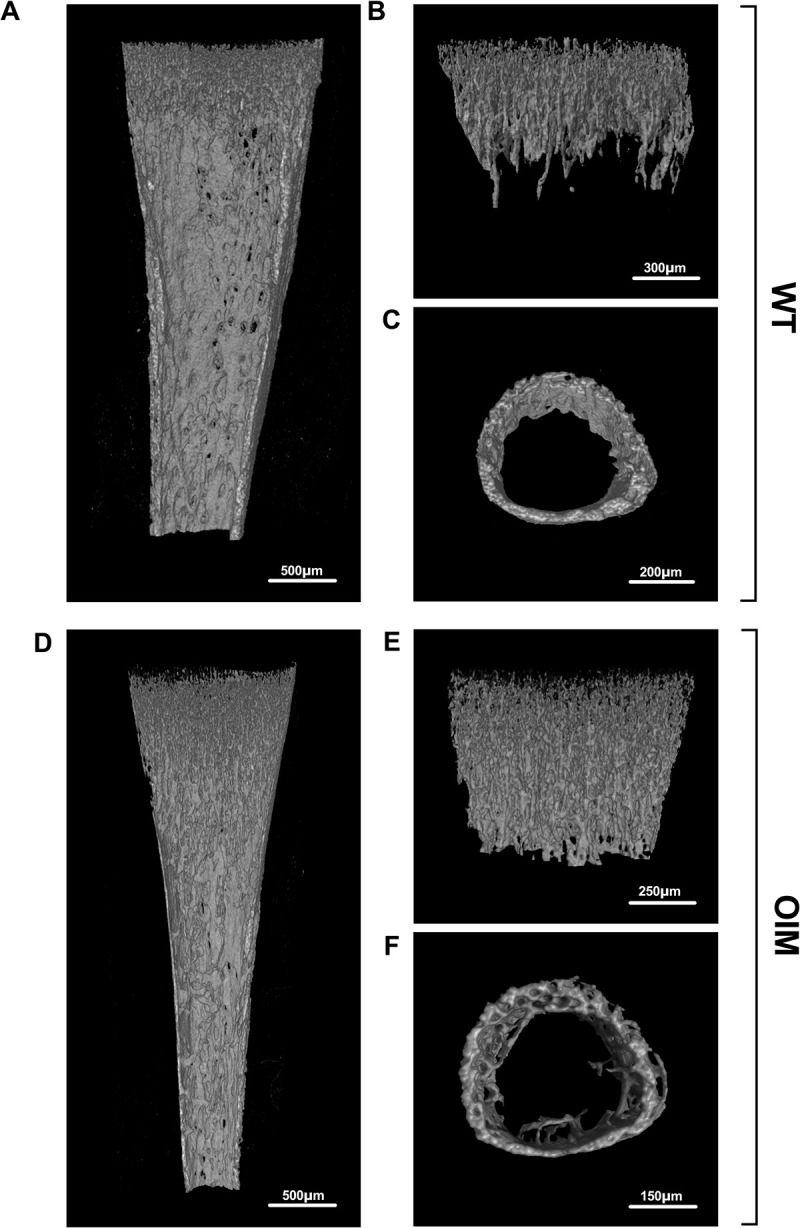
X-ray microCT imaging of the proximal tibia from 7 days old mice. **(A, D)** Representative sagittal sections of the proximal tibia showing the trabecular and cortical bone architecture. **(B, E)** Segmented view of the trabecular bone at proximal metaphysis obtained from 7-day old mice (WT, top and OIM, bottom). **(C, F)** Representative microCT image of the cortical bone at mid-diaphysis.

**Fig 2:**
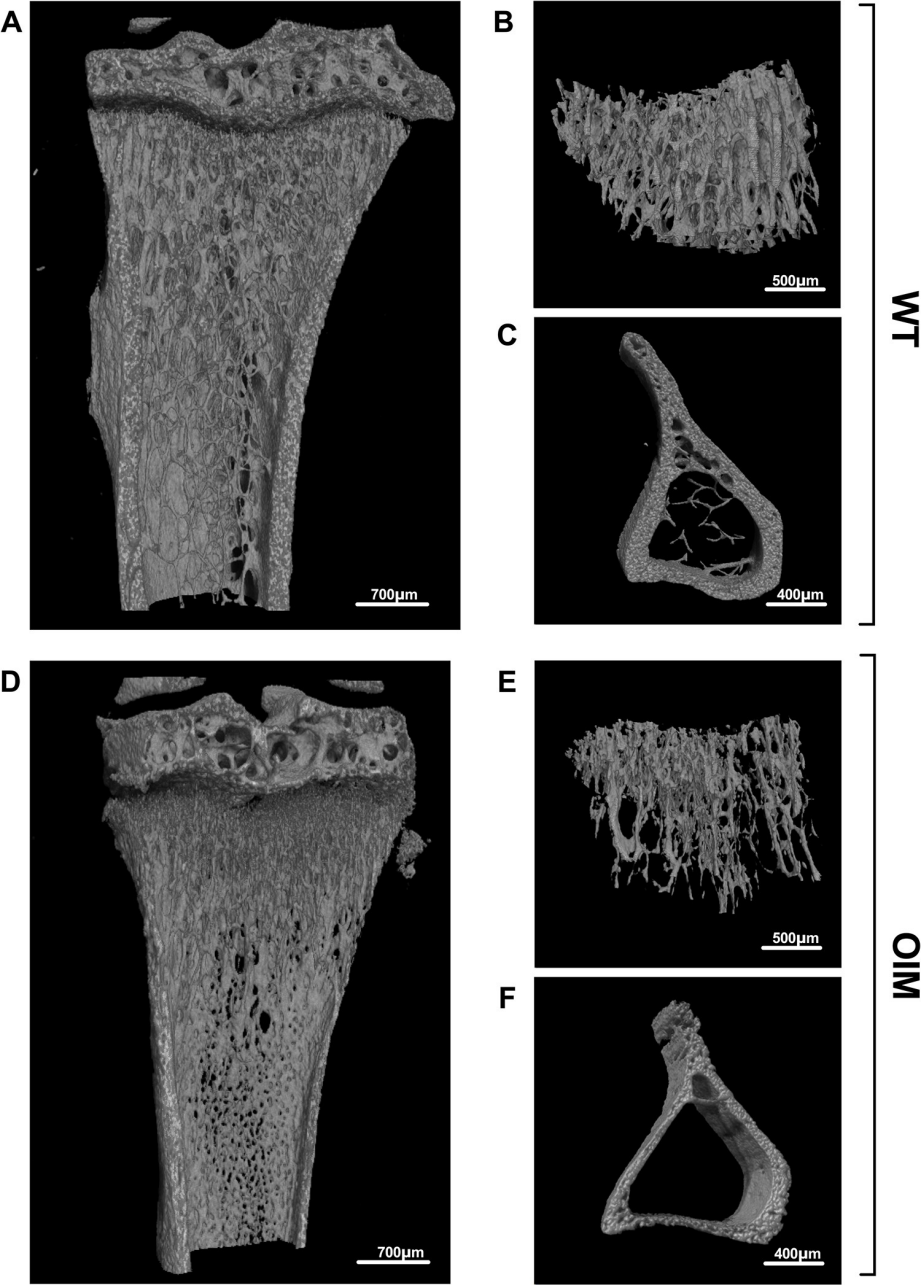
X-ray microCT imaging of the proximal tibia from 8 weeks-old mice. **(A, D)** Representative sagittal sections of the proximal tibia showing the trabecular and cortical bone architecture. **(B, E)** Segmented view of the trabecular bone obtained from 8-week-old mice (WT, top OIM, bottom). **(C, F)** Representative microCT image of the cortical bone at mid-diaphysis.

Comparative microCT analysis between OIM and WT shows that 1-week-old OIM mice had increased trabecular bone volume, with trabecular bone extending along the medullary canal more than in WT. In addition, the medullary canal thickness of 1-week old OIM is smaller than WT (**Figs 1 and 2**). In adult OIM mice, we observed the typical decrease in cortical thickness and trabecular network, which are the hallmark of the OIM phenotype.

**Fig 3:**
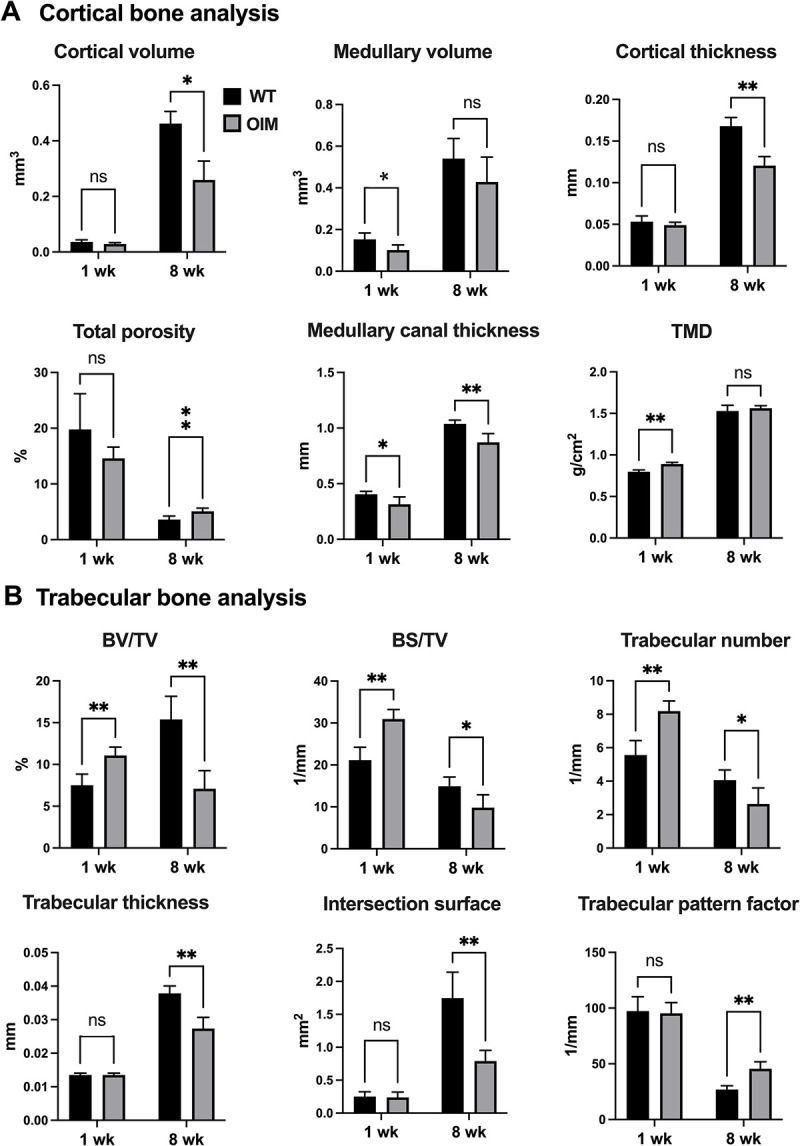
MicroCT morphometric analysis. **(A)** Quantification of morphometric parameters of tibial trabecular bone in 1 week-old and in 8 week-old WT and OIM mice. Cortical volume (mm^3^), medullary volume (mm^3^), cortical thickness (mm), total porosity (%), medullary canal thickness (mm), tissue mineral density (TMD, g/cm^2^). **(B)** Quantification of morphometric parameters of tibial cortical bone. Bone volume percent (BV/TV, %), bone surface density (BS/TV, 1/mm), trabecular number (1/mm), trabecular thickness (mm), Intersection surface (mm^2^), trabecular pattern factor (1/mm). All microCT parameters were analysed using Bonferroni’s multiple comparison post hoc test., ***P < 0.001, **P < 0.01 and * P < 0.05. ns: not significant. n = 7 for all the groups.

Compared to their WT counterparts, proximal tibia analysis of 1-week-old OIM revealed a significant decrease in medullary (or marrow) canal thickness (which measures the diameter of the medullary canal) i.e., 0.40±0.01 mm vs 0.33±0.02 mm, WT vs OIM, mean±SEM, n = 7, P<0.01), a significant increase in TMD (tissue mineral density) i.e., 0.80±0.10 g/cm^2^ vs 0.89±0.01 g/cm^2^, n = 7, P<0.0001), and a significant decrease in medullary volume i.e., 0.15±0.01 mm^3^ vs 0.10±0.01 mm^3^, n = 7, P<0.01), with no significant differences for cortical volume, cortical thickness and total porosity (**Figs 1** and **3A**). We observed no significant difference for trabecular thickness, intersection surface (which is the surface of the volume of interest intersected by solid binarized objects) and trabecular pattern factor (which relates to the arrangement and connectivity of trabeculae within bone) (**Figs 1** and **3B**). However, OIM bones showed a significant increase in BV/TV (which corresponds to the bone volume fraction, calculated by the ratio of the segmented bone volume to the total volume of the region of interest) i.e., 7.52±0.5% vs 11.08±0.37%, WT vs OIM, n = 7, P<0.0001), in BS/TV (which quantifies the bone surface density, as the ratio of the segmented bone surface to the total volume of the region of interest) i.e., 21.13±1.16 mm^-1^ vs 30.96±0.86 mm^-1^, n = 7, P<0.0001) and in trabecular number (5.57±0.32 mm^-1^ vs 8.19±0.23 mm^-1^, n = 7, P<0.0001).

In contrast to what observed in 1-week-old mice, we noted in 8-week-old OIM mice a decrease in cortical bone volume (0.43±0.02 mm^3^ vs 0.26±0.03 mm^3^,WT vs OIM, n = 7, P<0.0001), cortical thickness (0.17±0.01 mm vs 0.12±0.01 mm, n = 7, P<0.0001), medullary canal thickness (1.04±0.01 mm vs 0.88±0.03 mm, n = 7, P<0.001), BV/TV (15.40±1.13 vs 7.10±0.88, n = 7, P<0.001), BS/TV (14.90±0.90 1/mm vs 9.81±1.25 1/mm, n = 7, P<0.01), trabecular number (4.06±0.25 1/mm vs 2.64±0.39 1/mm, n = 7, P<0.01), trabecular thickness (0.04±0.00 mm vs 0.03±0.00 mm, n = 7, P<0.0001), intersection surface (1.75±0.16 mm^2^ vs 0.79±0.06 mm^2^, n = 7, P<0.001), and an increase in total bone porosity (3.61±0.25% vs 5.10±0.23%, n = 7, P<0.001). In addition, as we previously reported, we observed an increase in trabecular pattern factor (26.92±1.40 mm^-1^ vs 45.62±2.52 mm^-1^, n = 7, P<0.0001), indicative of a less interconnected and structurally sound trabecular network, which is associated with lower bone strength and compromised bone integrity.

### *In vitro* mineralization deposition by OIM osteoblasts is abundant but disorganized

**Fig 4:**
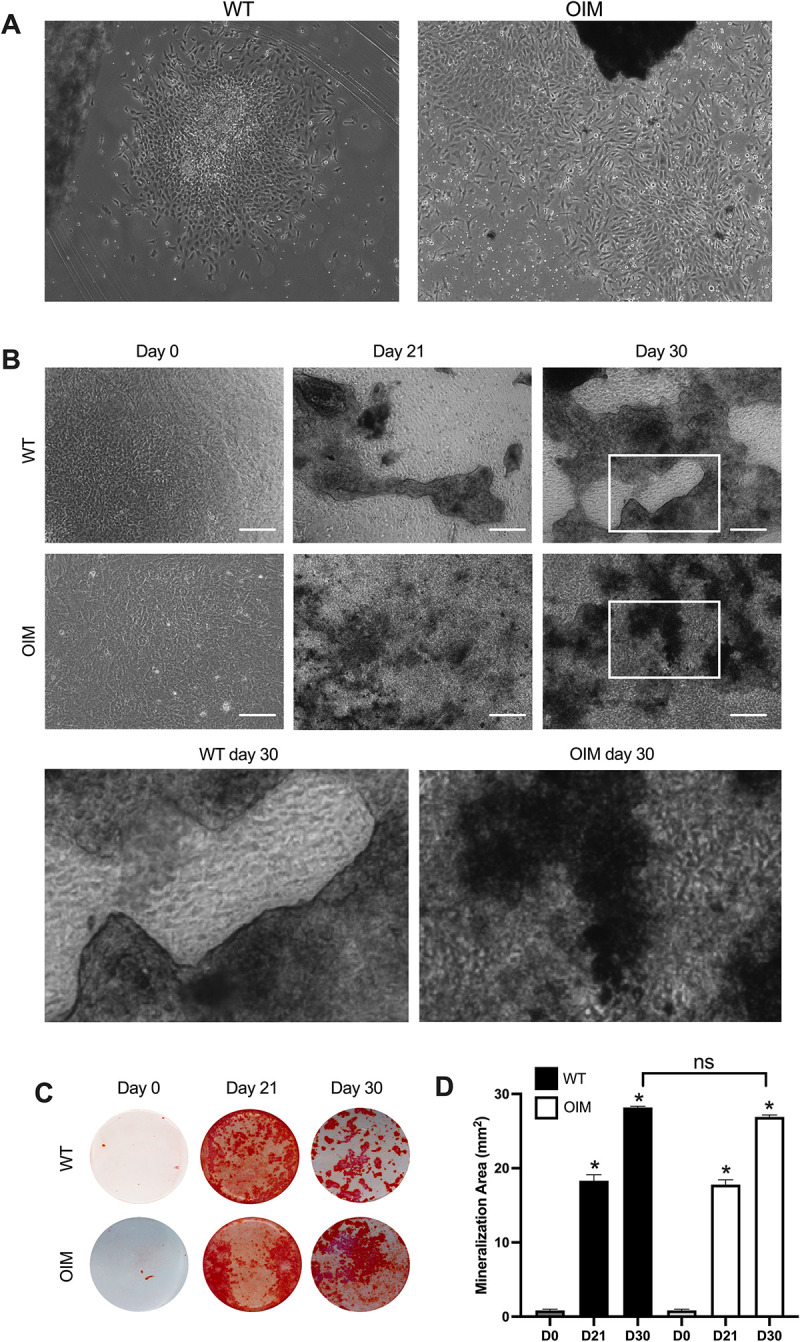
*In vitro* assessment of the mineral deposition in WT and OIM osteoblasts. **(A)** Phase contrast of primary calvaria osteoblasts. **(B)** Phase contrast of osteoblasts cultured in basal and osteogenic conditions at day 0, 21, and 30. **(C)** Alizarin Red staining. **(D)** Measurement of the mineralization area.

Primary pre-osteoblasts isolated from the calvaria of WT and OIM neonates, were expanded in non-osteogenic media (**Fig 4A**). Upon reaching near confluence, their expansion medium was replaced by osteogenic inductive medium, and a 30-day differentiation period commenced, during which medium was replaced three times per week (**Fig 4B**). Both cell types adopted the typical cobblestone morphology, which was qualitatively visible from as early as day 21, whereby WT cells deposited minerals in a very structured manner, forming spicules with well-defined thick borders (**Fig 4B**). By day 30, these spicules became interconnected to form a network of well-organised mineral matrix. In contrast, OIM cells produced an abundant amount of minerals that failed to organize. Instead, they remained diffuse throughout the dish and did not form clear mineralization spicules with well-defined borders. Alizarin red staining, which detects the presence of calcium deposits in a mineralized matrix, confirmed the formation of mineralized spicules by WT osteoblasts and the decreased organized deposition of calcium by OIM cells (**Fig 4C**), despite the total amount of minerals not significantly differing (**Fig 4D**).

### Transcriptome analysis reveals that the OIM-causative variant affects the expression of multiple genes across various pathways, including osteogenesis, cell stress response, TGF-β and autophagy, during osteogenic differentiation, (OIM d21 vs OIM d0) compared to (WT d21 vs WT d0)

In OIM cells, the recessive *Col1a2* variant affects the reading frame and resulted in an incorrect pro-peptide and a new stop codon [4]. This leads to the intracellular accumulation of a defective *Col1a2* mRNA transcript (non-sense mediated decay escape) that is not translated into Co1a2 procollagen molecule. In the bone extracellular matrix (ECM), this results in bone tissue accumulation of a dysfunctional type I collagen homotrimer a1(I)_3_, in replacement of the wild type a1(I)_2_a2(I)_1_ heterotrimer [15].

**Fig 5:**
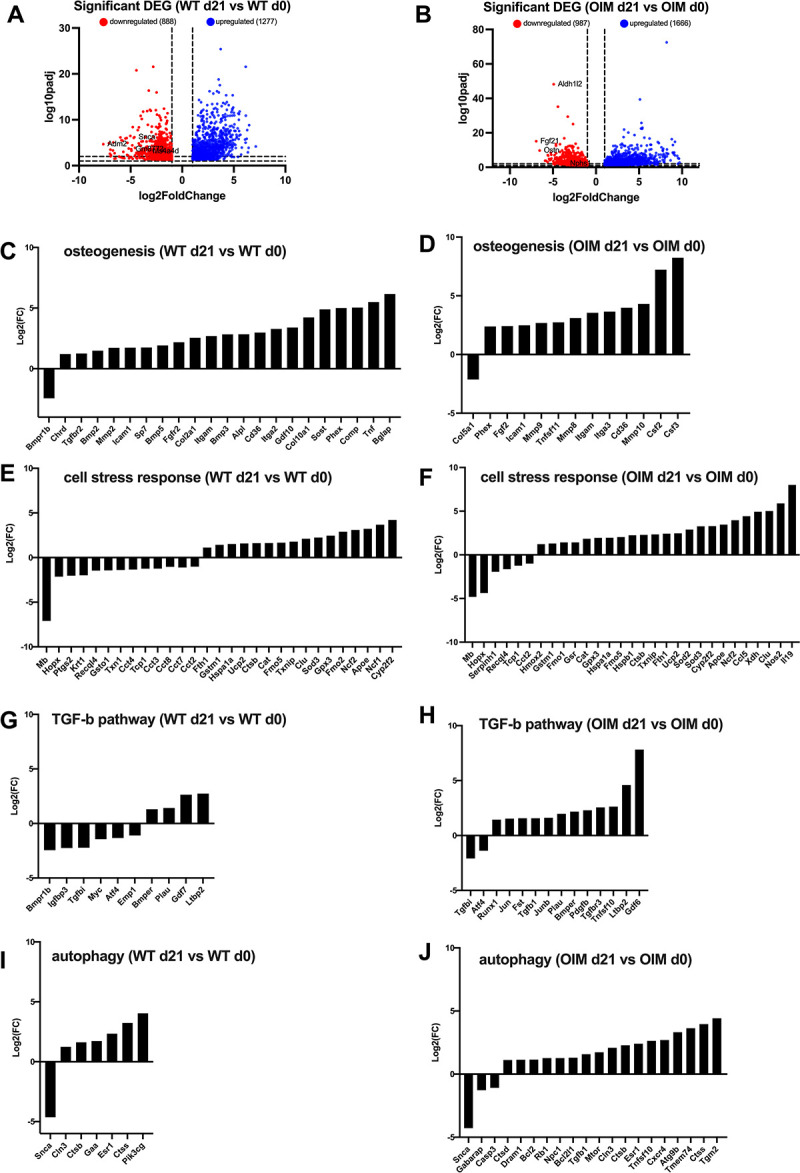
RNAseq analysis of mature WT osteoblasts vs WT pre-osteoblasts (WT d21 vs WT d0) and mature OIM osteoblasts vs OIM pre-osteoblasts (OIM d21 vs OIM d0) during osteogenic differentiation **(A)** Volcano plots showing significant (FDR adjusted p<0.05) DEGs (differentially expressed genes) in (WT d21 vs WT d0) and(OIM d21 vs OIM d0) **(B)** after 3 weeks in osteogenic induction medium. DEGs were plotted by pathways: osteogenesis **(C, D)**, cell stress response **(E, F)**, TGF-Beta pathway **(G, H)**, autophagy **(I, J)**.

We first compared the response of WT and OIM cells to 21 days of *in vitro* osteogenic induction (d0 vs d21 for both WT and OIM) to determine the impact of the OIM-causative *Col1a2* variant on the whole genome. Results showed that osteogenic induction is associated to the downregulation of 888 genes and the upregulation of 1277 genes in WT cells (**Fig 5A**), and to the downregulation of 987 genes and the upregulation of 1666 genes in OIM cells (**Fig 5B**).

More precisely, in WT cells, 21 genes involved in osteogenesis are up-regulated (WTd21 vs WTd0), (*Chrd*, *Tgfbr2*, *Bmp2*, *Mmp2*, *Icam1*, *Sp7*, *Bmp5*, *Fgfr2*, *Col2a1*, *Itgam*, *Bmp3*, *Alpl*, *Cd36*, *Itga2*, *Gdf10*, *Col10a1*, *Sost*, *Phex*, *Comp*, *Tnf*, *and Bglap*, fold-change for each gene is given in **S1 Table**), and 1 is down-regulated (*Bmpr1b*) (**Fig 5C**) whilst in OIM cells OIMd0 vs OIMd21), 12 genes are up-regulated (*Phex*, *Fgf2*, *Icam1*, *Mmp9*, *Tnfsf11*, *Mmp8*, *Itgam*, *Itga3*, *Cd36*, *Mmp10*, *Csf2 and Csf3*) and 1 is down-regulated (*Col5a1*) in OIM cells (**Fig 5D**). Among those, only 4 genes are upregulated in both WT and OIM cells, i.e. *Icam1*, *Itgam*, *Cd36* and *Phex*. The key interesting genes that are strongly up-regulated only in WT cells include *Alpl* (alkaline phosphatase, 7.2-fold increase), *Bglap* (71.7-fold increase), *Sost* (29.8-fold increase), *Fgfr2* (4.5-fold increase), *Sp7* (3.4-fold increase) and *Tnf* (45.1-fold increase). Conversely, key genes strongly up-regulated only in OIM cells include Mmp9 (6.4-fold increase), *Mmp8* (8.6-fold increase), *Mmp10* (19.8-fold increase), *Tnfs11* (6.7-fold increase), *Csf2* (149-fold increase), and *Csf3* (301-fold increase).

We found that 16 genes involved in the cell stress response pathway are up-regulated in WT cells (*Fth1*, *Gstm1*, *Hspa1a*, *Ucp2*, *Ctsb*, *Cat*, *Fmo5*, *Txnip*, *Clu*, *Sod3*, *Gpx3*, *Fmo2*, *Ncf2*, *Apoe*, *Ncf1*, *Cyp2f2*) and 13 are down-regulated (*Mb*, *Hopx*, *Ptgs2*, *Krt1*, *Recql4*, *Gsto1*, *Txn1*, *Cct4*, *Tcp1*, *Cct3*, *Cct7*, *Cct8*, *Cct2*) (**Fig 5E**). In OIM cells, 14 out of the 16 gene up-regulated in WT cells are also up-regulated (except for *Fmo2* and *Ncf1*) (**Fig 5F**). An additional 9 genes are also up-regulated (*Hmox2*, *Fmo1*, *Gsr*, *Hspb1*, *Sod2*, *Ccl5*, *Xdh*, *Nos2*, *Il19*), with *Il19*, *Nos2*, *Clu* and *Xdh* being the most up-regulated (256.8, 59.3, 32.2, and 30.4-fold increase, respectively). Only 6 genes are downregulated in OIM cells (*Mb*, *Hopx*, *Serpinh1*, *Recql4*, *Tcp1*, *Cct2*) with *Mb*, *Hopx*, *Recql4 and Tcp1* being down-regulated in both OIM and WT cells.

In WT cells, only 4 genes involved in the TGF-β pathway are up-regulated (*Bmper*, *Plau*, *Gdf7 and Ltbp2*) and 6 are down-regulated (*Bmpr1b*, *Igfbp3*, *Tgfbi*, *Myc*, *Atf4*, *Emp1*) (**Fig 5G**), whilst in OIM cells, only *Tgfbi* and *Atf4* are down-regulated and 12 genes are up-regulated (*Runx1*, *Jun*, *Fst*, *Tgfb1*, *Junb*, *Plau*, *Bmper*, *Pdgfb*, *Tgfbr3*, *Tnfsf10*, *Ltbp2*, *Gdf6*) (**Fig 5H**). Among those, *Gdf6* and *Ltbp2* are the most up-regulated in OIM cells (226.4 and 24.1-fold increase, respectively).

We observed that 6 genes involved in the autophagy pathway are up-regulated in WT cells (*Cln3*, *Ctsb*, *Gaa*, *Esr1*, *Ctss*, *Pik3cg*) and 1 gene is down-regulated (*Snca*) (**Fig 5I**), whilst in OIM cells 3 genes are down-regulated (*Snca*, *Gabarap*, *Casp3*) and 17 genes are upregulated (*Ctsd*, *Dram1*, *Bcl2*, *Rb1*, *Npc1*, *Bcl2l1*, *Tgfb1*, *Mtor*, *Cln3*, *Ctsb*, *Esr1*, *Tnfsf10*, *Cxcr4*, *Atg9b*, *Tmem74*, *Ctss*, *Tgm2*) (**Fig 5J**).

### Comparative analysis of differentiated OIM and WT cells show that the OIM-causative variant compromises osteoblast maturation

**Fig 6:**
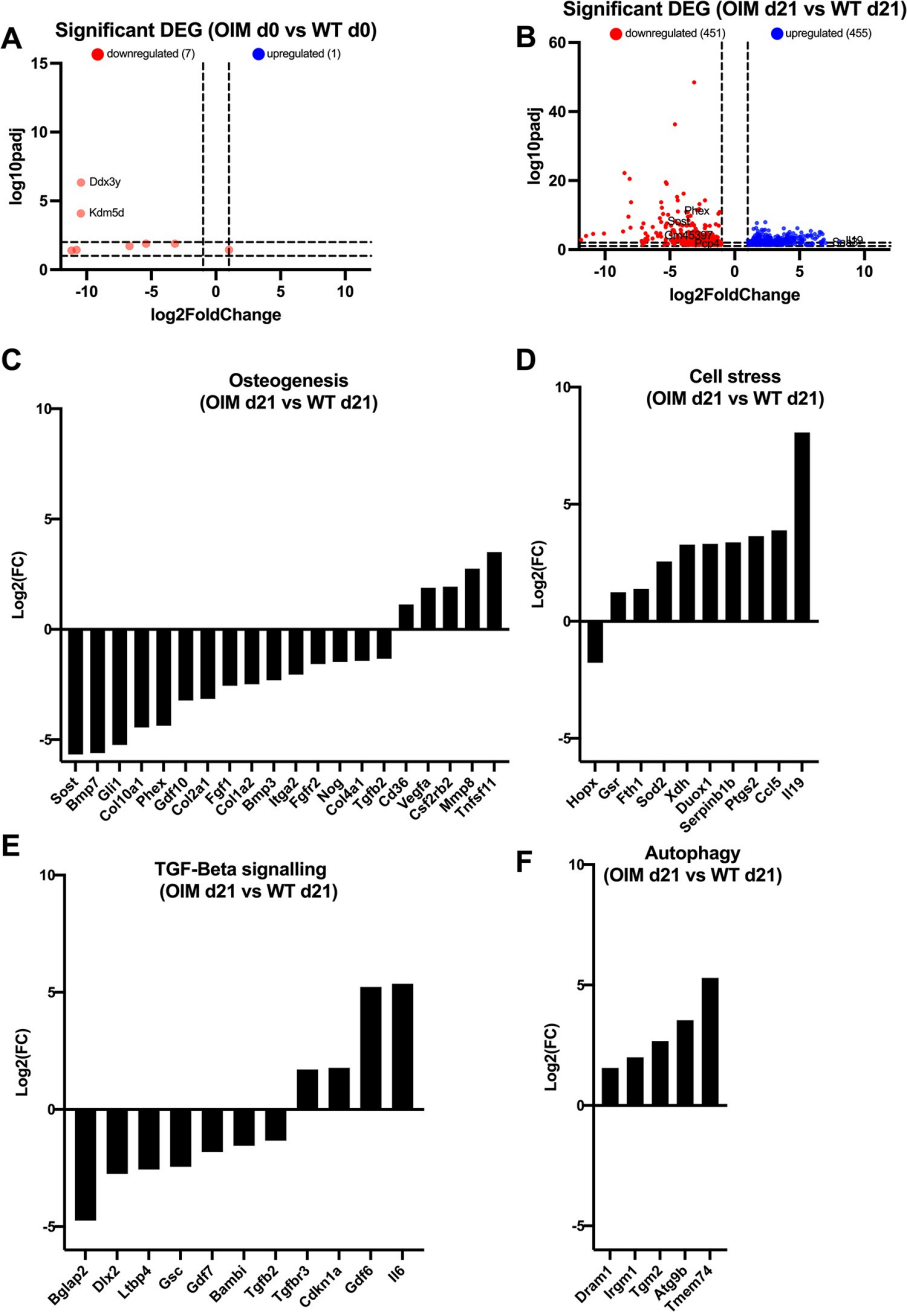
RNAseq analysis of mature WT vs mature OIM osteoblasts (OIM d21 vs WT d21) and WT pre-osteoblasts vs OIM pre osteoblasts (OIM d0 vs WT d0). **(A)** Volcano plots showing significant (FDR adjusted p<0.05) DEGs (differentially expressed genes) in OIM d21 vs WT d21 **(B)** OIM d0 vs WT d0. DEGs were plotted by pathways: TGF-β signalling **(C)**, cell stress **(D)**, osteogenesis **(E)**, autophagy **(F)**.

We next compare the transcriptome of OIM and WT cells following 21 days of culture in osteogenic permissive condition to determine the impact of the OI-causative variant on osteoblast phenotype (**Fig 6**).

Undifferentiated OIM and WT cells show similar transcriptome media (**Fig 6A**). In contrast, following osteogenic differentiation, gene expression differs significantly, with 451 genes being downregulated and 455 genes being upregulated in OIM, when compared to WT. These data further confirm that the OI-causative variant impacts the gene expression profile of differentiated osteoblasts (**Fig 6B**).

When comparing the level of expression of genes expressed in both OIM and WT differentiated cells, results show that 15 genes involved I the osteogenic pathway show lower expression in OIM cells (*Sost*, *Bmp7*, *Gli1*, *Col10a1*, *Phex*, *Gdf10*, *Col2a1*, *Fgf1*, *Col1a2*, *Bmp3*, *Itga2*, *Fgfr2*, *Nog*, *Col4a1*, *Tgfb2*), and 5 showed lower expression (*Sost*, *Bmp7*, *Gli*, *Col10a1 and Phex*) (**Fig 6C**). For the cell stress pathway, 9 genes showed higher expression (*Gsr*, *Fth1*, *Sod2*, *Xdh*, *Duox1*, *Serpinb1b*, *Ptgs2*, *Ccl5*, *Il19*) and1 a lower expression (*Hopx*) (**Fig 6D**). For the TGF-β pathway, 4 genes showed higher expression in OIM cells (*Tgfbr3*, *Cdkn1a*, *Gdf6 and Il6*, 3.3, 3.4, 37.5 and 41.1-fold increase in OIM cells compared to WT cells, respectively) and 7 showed lower expression (*Bglap2*, *Dlx2*, *Ltbp4*, *Gsc*, *Gdf7*, *Bambi*, *Tgfb2*), with *Bglap2* being the less expressed (26.7-fold decreased in OIM cells) (**Fig 6E**). For the autophagy pathway, 5 genes showed higher expression in OIM cells (*Dram1*, *Irgm1*, *Tgm2*, *Atg9b*, *Tmem74*), with *Atg9b* and *Tmem74* showing the highest expressions (11.6 and 39.2-fold increase, respectively).

## Discussion

The integrity of the skeletal system is maintained through the constant remodelling of the bone extracellular matrix (ECM) by osteoblasts, through the synthesis of type I collagen and minerals, and by osteoclasts, which are responsible for bone resorption and release of minerals back into the bloodstream. The presence of the recessive OIM-causative variant directly results in the extracellular secretion of abnormal homotrimeric collagen type I and intracellularly to the accumulation of *Col1a2* mRNA. These two primary effects contribute to abnormal osteoblast development and function, and dysregulated bone remodelling leading to skeletal fragility to homozygous OIM mice. The comparative imaging of 1-week and 8-week-old OIM and WT tibia revealed that by one week of age, several bone parameters are not yet impacted by the recessive OIM-causative variants. This includes (1) the volume of the dense and compact bone (also called cortical bone) which function is to protect the inner trabecular bone and to provide strength and support to the skeleton; (2) the volume of the bone marrow cavity (medullary canal), which influence the bone marrow’s capacity to produce blood cells; (3) the thickness of the cortex, which contributes to the overall strength of bones by providing the ability to withstand mechanical stress and resistance to fractures; (4) the total porosity, which refers to the volume of void space within the bone tissue; (5) the thickness of the trabecular bone, which contributes to the bone’s resistance to fractures, in particular in the regions of high load-bearing, (6) the “intersection surface”, which refers to the surface where trabeculae cross each other to form a three-dimensional lattice-like structure that confers mechanical strength to the trabecular bone by allowing the distribution of forces within the bone, and finally (7) the “trabecular pattern factor”, which gives an overall measure of the bone quality. However, 1-week-old OIM mouse bones already show a decrease in the thickness of the medullary canal, and an increase in the bone volume fraction (BV/TV), bone surface density (BS/TV), trabecular number, and tissue mineral density (TMD). This is possibly due to the visible greater size of the OIM growth plate compared to age-matched WT bones, which is likely a compensatory mechanism in response to the abnormal structure of the collagen matrix. By the age of 8 weeks, skeletal maturity and growth plate closure is almost complete. All parameters except for medullary volume, such as BV/TV, BS/TV and trabecular number demonstrate deterioration by this stage, reflecting an overall picture of reduced trabecular bone.

Together, these results suggests that perinatal interventions could protect the developing OIM bones. The analysis of mineral deposition by OIM and WT osteoblasts showed that the OIM-causative mutation does not impact the quantity of minerals being deposited in the bone matrix, but rather its organization. This is important because mineralization of the collagen network (which provides tensile strength) confers compressive strength to bones and thereby contributes to resistance to fracture. This could be due to impaired differentiation of OIM pre-osteoblasts.

Transcriptome analysis revealed that OIM pre-osteoblasts isolated from neonatal calvaria do not differ from their WT counterparts when cultivated in expansion media. However, when exposed to osteogenic permissive conditions over a 3-week period, transcriptome profiling revealed that, OIM cells activate different signalling pathways compared to WT pre-osteoblasts. As previously reported, incomplete osteogenesis was found in OIM cells, with 17 genes being exclusively upregulated in WT osteoblasts (*Tgfbr2*, *Bmp2*, *Mmp2*, *Sp7*, *Bmp5*, *Fgfr2*, *Col2a1*, *Bmp3*, *Fdg10*, *Comp*, *Itga2*, *Tnf*, *Sost*, *Alp*, *Bglap*, *Col10a1*) and 10 genes being exclusively upregulated in OIM osteoblasts (*Tgfb1*, *Fgf2*, *Tgfbr3*, *Mmp9*, *Itga3*, *Tnfsf11*, *Mmp8*, *Mmp10*, *Csf2 and Csf3*), whilst only 2 genes are exclusively downregulated in OIM osteoblasts (*ColL5a1* and *Serpinh1*), and one gene in WT osteoblasts (*Bmpr1b*).

WT cells, unlike OIM cells, express key genes involved in osteoblast maturation and matrix mineralisation. Those include for example *Sost* (Sclerostin) which regulates bone turnover by inhibiting early osteoblast activity [16], and *Sp7* which expression is promoted by *Fgfr2* (Fibroblast Growth Factor Receptor 2) [17]. Additional genes include *Sp7* (Osterix) which stimulates the maturation of osteoblasts and plays a critical role in bone matrix mineralization through the activation of *Bglap* (Osteocalcin) which is itself involved in calcium ion deposition and matrix hardening, and *Alpl*, which is involved in the formation of hydroxyapatite crystals. Finally, *Tnf* (Tumour Necrosis Factor) influences osteogenesis by regulating bone turnover and stimulating osteoblasts to produce factors such as VEGF (Vascular endothelial growth factor). Unlike WT cells, genes that are up-regulated only in OIM cells are involved in collagen degradation (*Mmp8* and *Mmp10*, Matrix Metallo Proteinases 8 and 10) [18], bone resorption, i.e. *Mmp9* [19] and *Tnfsf11 (*tumour necrosis factor ligand superfamily 11, also called *Rankl* or receptor activator of nuclear factor kappa-B ligand) [20], and osteoclastogenesis (*Csf2* and *3*, Colony stimulating factor 2 and 3). Although *Phex* (phosphate regulating neutral endo peptidase) is up-regulated in both WT and OIM, up-regulation is markedly greater in WT (32.1-fold increase in WT compared to 5.2-fold increase in OIM). These data further suggest that bone mineralization is impaired in OIM, since PHEX regulates phosphate levels in the body, which is essential for the formation of hydroxyapatites [21]. *Serpinh1* (collagen binding protein HSP47), which plays a crucial role in the proper folding and stabilization of collagen molecules by binding to newly synthesized collagen chains in the endoplasmic reticulum (ER) [22], is downregulated in OIM cells, suggesting that homotrimer type I collagen’s stability and functionality may be compromised in the ECM. Interestingly, we also observed that *Gdf6* (Growth differentiation factor 6, also called *Bmp13*, bone morphogenic protein 13), which is a potent inhibitor of osteoblast maturation [23], is strongly upregulated in OIM cells but not in WT cells. *Bmpr1b* (bone morphogenic protein receptor 1b), which is downregulated in WT cells, but not in OIM, ensures the proper commitment to osteogenesis and is involved in the transition to a mature osteoblast stage, as mature osteoblast do not depend on BMP signalling in the same way as progenitor cells. Conversely *Gdf7* (Growth differentiation factor 7, also called *Bmp12*), which is only up-regulated in WT, induces the expression of osteogenic key genes involved in the mineralization of the bone matrix [24], such as *Bglap* and *Alp*. The upregulation of *Pdgfb* (platelet derived growth factor B) and *Junb* (JunB proto-oncogene) further suggest the proliferation of OIM osteoprogenitors [25, 26].

Excessive TGF-β (transforming growth factor beta) signalling has previously been shown to be a common mechanism of OI in both recessive and dominant OI mouse models [8, 9]. The abnormal bone ECM structure prevents the proper decorin-mediated sequestration of latent TGF-β in the bone matrix, which results in excessive release of TGF-β in its active form [8]. The upregulation of the TGF-β pathway in OIM cells was evidenced by the increase in the expression of *Tgfb1* and *Tgfbr3* (transforming growth factor beta receptor 3) which are both involved in the recruitment of endogenous MSCs and the proliferation of osteoprogenitor cells [27]. TGFB3 promotes osteoprogenitor cell proliferation through the activation of non-Smad pathways such as MAPK and P13K, which stimulate the production of growth factors, but is also secreted by osteoprogenitors themselves in an autocrine manner. *Il6* (Interleukin 6) which contributes to bone resorption through the stimulation of osteoclast activity [28] is also higher in OIM cells.

These data collectively suggest that in OIM cells, both osteogenesis and bone ECM mineralization are compromised, with OIM cells not reaching the same level of maturity as WT osteoblasts and remaining at an earlier stage of differentiation, while bone resorption and osteoclastogenesis are stimulated. The phenotype of OIM osteoblasts may have a feedback inhibitory effect on osteogenesis and on bone extracellular matrix structure by influencing the deposition of minerals and the organization of type I collagen fibres.

In OIM cells, we found an up-regulation of genes involved in the production of reactive oxygen species (ROS), such as *Xdh* (xanthine dehydrogenase) [29], which causes oxidative stress and inflammation in response to defective mRNA and/or the accumulation of dysfunctional proteins [30]. Other strongly up-regulated genes included *Nos2* (nitric oxide synthase 2), which is known to be induced in response to inflammatory signals as well as *Tnfsf10* (tumour necrosis factor related apoptosis inducing ligand), which acts as a pro-apoptotic and pro-autophagic protein during cellular stress [31]. We also observed the upregulation of *Rb1* (retinoblastoma 1), which plays a role in regulating apoptosis [32]. Activation of the autophagy pathway in OIM is also suggested by the upregulation of *Dram1* (damage-regulated autophagy modulator 1), *Atg9b* (autophagy related protein 9b) and *Tgm2* (trans glutaminase 2), the expression of which is induced by oxidative stress and which contributes to the initiation of autophagy by promoting the formation and expansion of the autophagosome [33–35]. *Cxcr4* (C-X-C chemokine receptor type 4), which is part of the cellular stress response and which expression has been shown to increase autophagy activity [36], is also upregulated. Activation of autophagy in OIM cells is also suggested by the upregulation of *Tmem74* (transmembrane 74), which promotes autophagy [37].

OIM cells may simultaneously be protected from oxidative damage, as suggested by the upregulation of *Gsr* (glutathione reductase), which plays a vital role in maintaining the cellular antioxidant defence mechanisms [38], and *Hmox2* (heme oxygenase 2), which protects cells against oxidative stress and inflammation through its involvement in heme degradation and subsequent bilirubin-mediated neutralization of ROS [39]. OIM cell protection against ROS-induced intra-cellular damage is also suggested by the upregulation of *Sod2* (superoxide dismutase 2), a mitochondrial enzyme that maintains the cellular redox balance by scavenging superoxide radicals and thereby preventing apoptosis triggered by excessive ROS [40]. Promotion of OIM cell survival as a defence mechanism against oxidative stress-induced apoptosis is also suggested by the upregulation of genes from the Bcl-2 family, which are involved in the regulation of apoptosis. For example, we observed an upregulation of *Bcl2* (B cell lymphoma 2), and *Bcl2l1* (B-cell lymphoma 2-like 1), which promote cell survival by preventing the degradation of various cellular components, and by decreasing autophagy through the inhibition of *Becn1* (beclin-1), concomitant with the downregulation of *Casp3* (caspase 3), a key mediator of apoptosis [41]. Together, these data suggest that the activation of the pro-apoptotic and pro-autophagic pathways in OIM cells may act as a protective mechanism against oxidative stress and other cellular stressors. Those may include the intracellular accumulation of untranslated Col1a2 mRNA, which disrupts cellular homeostasis [42], affecting mitochondrial function and leading to impaired energy production and increased ROS production.

This is the first study showing how the OIM-causative mutation impacts the whole genome during the process of osteogenic differentiation and compares the transcriptome of OIM and WT pre-osteoblasts and osteoblasts. he fact that OIM fail to fully mature but remain at an earlier stage of differentiation, has several consequences, not least the activation of the osteoclast pathway, thus compromising bone remodelling by favouring bone resorption. It also negatively impacts the bone ECM mineralization process (size, distribution, and organization of minerals), thereby contributing to further decreasing bone strength, especially as the structure of the collagen network may already contribute to defective matrix mineralization. In addition, the lower levels of osteocalcin in OIM may have knock-on effects on overall health, since this non-collagenous protein is not only involved in the process of mineralization through its incorporation into the bone matrix and its involvement in the process of hydroxyapatite crystals formation but is also involved in the regulation of calcium homeostasis, glucose metabolism, insulin sensitivity, and metabolism. Our data indicate that the primary intracellular (COL1A2 mRNA accumulation) and extracellular (homotrimeric COL1 lattice) consequences of the homozygous OIM-causative variant trigger a cascade of knock-on effects, both at the level of osteoblast behaviour and matrix structural properties, that together contribute to bone brittleness in a multifactorial manner.

**Fig 7:**
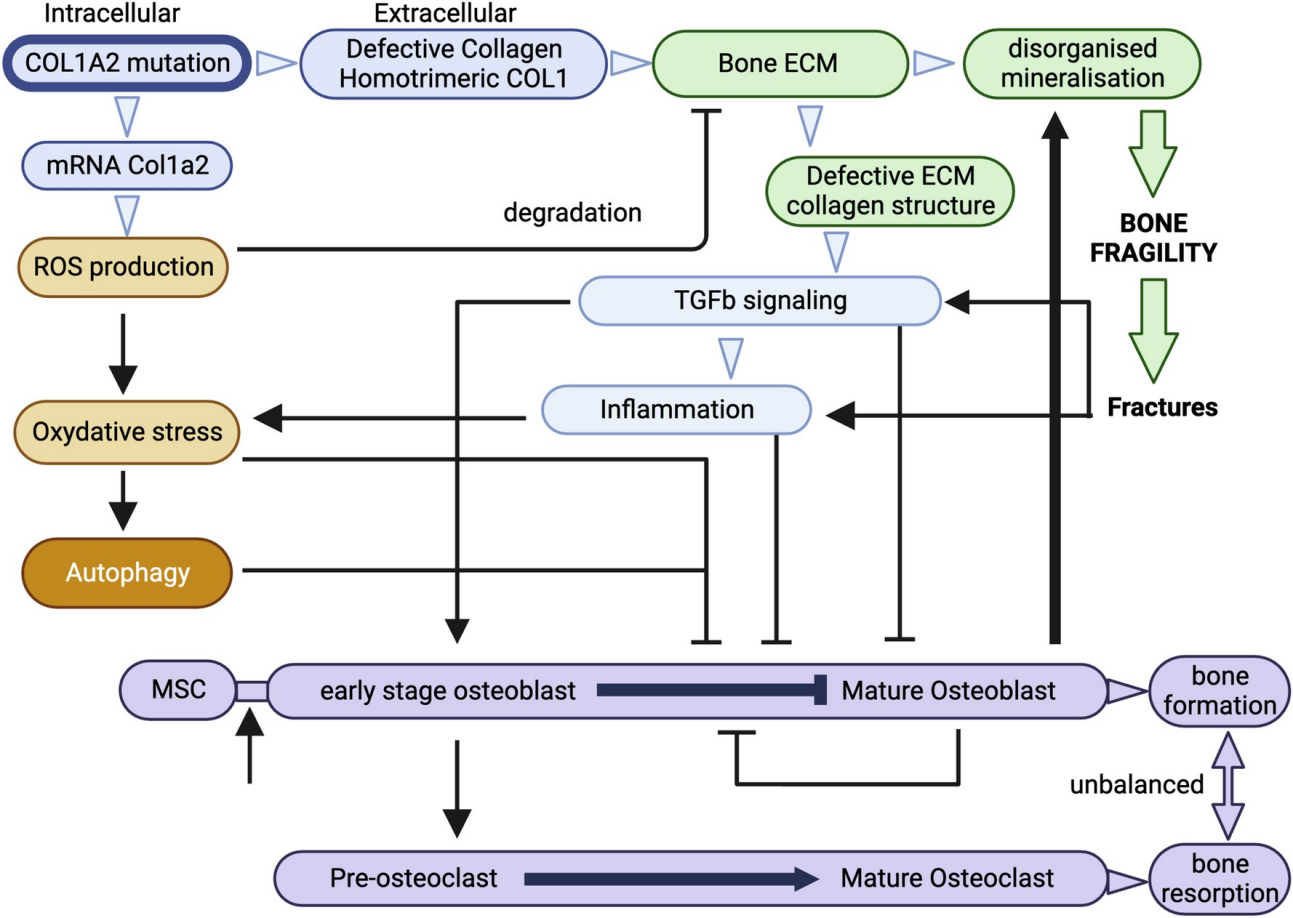
Simplified model of the various pathways contributing to bone fragility in homozygous OIM mice. Created by Biorender.

**Fig 7** shows a simplified overview of the directly responsible to.

These data provide an overview of the pleiotropic effects in the homozygous OIM mice and give additional insight into the mechanisms of OI disease progression in this model. Our data also identify multiple clinically relevant endpoints to assess the efficacy of innovative therapeutic intervention aiming at improving skeletal health for people with OI. However, these data elucidate the mechanisms of OI in this mouse model and may or may not directly translate to human.

## Supporting information

S1 Table R1: RNAseq analysis of mature WT osteoblasts vs WT pre-osteoblasts (WT d21 vs WT d0) (A), mature OIM osteoblasts vs OIM pre-osteoblasts (OIM d21 vs OIM d0), and mature OIM vs mature WT (OIM d21 vs WT 21). (DOCX)
