# Supplementary material for: Pleiotropic effects of a recessive Col1a2 mutation occurring in a mouse model of severe osteogenesis imperfecta
Source: PLoS One. 2025 Feb 5;20(2):e0309801. doi: 10.1371/journal.pone.0309801 (PMC11798436; doi:10.1371/journal.pone.0309801)
Supplement: S1 Table — (DOCX) [file pone.0309801.s001.docx]

**Supplementary Information**

RNAseq analysis of mature WT osteoblasts vs WT pre-osteoblasts (WT d21 vs WT d0) (A), mature OIM osteoblasts vs OIM pre-osteoblasts (OIM d21 vs OIM d0), and mature OIM vs mature WT (OIM d21 vs WT 21) .

**Table 1**

**A**

| **WT d21 vs WT d0** | | | | | | | |
| --- | --- | --- | --- | --- | --- | --- | --- |
| **osteogenesis** | | **Cell stress** | | **TGF-b** | | **autophagy** | |
| **gene name** | **fold change** | **gene name** | **fold change** | **gene name** | **fold change** | **gene name** | **fold change** |
| Bmpr1b | -5.5 | Mb | -137.1 | Bmpr1b | -5.5 | Snca | -24.9 |
| Chrd | 2.3 | Hopx | -4.4 | Igfbp3 | -4.8 | Cln3 | 2.4 |
| Tgfbr2 | 2.4 | Ptgs2 | -4.1 | Tgfbi | -4.7 | Ctsb | 3.1 |
| Bmp2 | 2.8 | Krt1 | -3.9 | Myc | -2.7 | Gaa | 3.3 |
| Mmp2 | 3.3 | Recql4 | -2.8 | Atf4 | -2.5 | Esr1 | 5.0 |
| Icam1 | 3.3 | Gsto1 | -2.7 | Emp1 | -2.1 | Ctss | 9.4 |
| Sp7 | 3.4 | Txn1 | -2.6 | Bmper | 2.5 | Pik3cg | 16.4 |
| Bmp5 | 3.8 | Cct4 | -2.5 | Plau | 2.7 |  |  |
| Fgfr2 | 4.5 | Tcp1 | -2.4 | Gdf7 | 6.2 |  |  |
| Col2a1 | 5.8 | Cct3 | -2.4 | Ltbp2 | 6.7 |  |  |
| Itgam | 6.5 | Cct7 | -2.2 |  |  |  |  |
| Bmp3 | 7.1 | Cct8 | -2.1 |  |  |  |  |
| Alpl | 7.2 | Cct2 | -2.0 |  |  |  |  |
| Cd36 | 7.9 | Fth1 | 2.2 |  |  |  |  |
| Itga2 | 9.7 | Gstm1 | 2.7 |  |  |  |  |
| Gdf10 | 10.5 | Hspa1a | 2.9 |  |  |  |  |
| Col10a1 | 18.8 | Ucp2 | 3.0 |  |  |  |  |
| Sost | 29.8 | Ctsb | 3.1 |  |  |  |  |
| Phex | 32.1 | Cat | 3.1 |  |  |  |  |
| Comp | 32.9 | Fmo5 | 3.2 |  |  |  |  |
| Tnf | 45.1 | Txnip | 3.4 |  |  |  |  |
| Bglap | 71.7 | Clu | 4.3 |  |  |  |  |
|  |  | Sod3 | 4.7 |  |  |  |  |
|  |  | Gpx3 | 5.5 |  |  |  |  |
|  |  | Fmo2 | 7.4 |  |  |  |  |
|  |  | Ncf2 | 8.6 |  |  |  |  |
|  |  | Apoe | 9.3 |  |  |  |  |
|  |  | Ncf1 | 12.8 |  |  |  |  |
|  |  | Cyp2f2 | 18.6 |  |  |  |  |

**B**

| **OIM d21 vs OIM d0** | | | | | | | |
| --- | --- | --- | --- | --- | --- | --- | --- |
| **osteogenesis** | | **Cell stress** | | **TGF-b** | | **autophagy** | |
| **gene name** | **fold change** | **gene name** | **fold change** | **gene name** | **fold change** | **gene name** | **fold change** |
| Col5a1 | -4.4 | Mb | -28.3 | Tgfbi | -4.2 | Snca | -19.5 |
| Phex | 5.2 | Hopx | -20.8 | Atf4 | -2.6 | Gabarap | -2.4 |
| Fgf2 | 5.3 | Serpinh1 | -3.8 | Runx1 | 2.7 | Casp3 | -2.1 |
| Icam1 | 5.6 | Recql4 | -3.1 | Jun | 2.9 | Ctsd | 2.2 |
| Mmp9 | 6.4 | Tcp1 | -2.4 | Fst | 3.0 | Dram1 | 2.2 |
| Tnfsf11 | 6.7 | Cct2 | -2.0 | Tgfb1 | 3.0 | Bcl2 | 2.2 |
| Mmp8 | 8.6 | Hmox2 | 2.3 | Junb | 3.1 | Rb1 | 2.4 |
| Itgam | 11.7 | Gstm1 | 2.5 | Plau | 3.9 | Npc1 | 2.4 |
| Itga3 | 12.6 | Fmo1 | 2.7 | Bmper | 4.5 | Bcl2l1 | 2.5 |
| Cd36 | 15.7 | Gsr | 2.7 | Pdgfb | 4.9 | Tgfb1 | 3.0 |
| Mmp10 | 19.8 | Cat | 3.6 | Tgfbr3 | 5.9 | Mtor | 3.3 |
| Csf2 | 149.0 | Gpx3 | 3.8 | Tnfsf10 | 6.2 | Cln3 | 4.2 |
| Csf3 | 301.2 | Hspa1a | 3.9 | Ltbp2 | 24.1 | Ctsb | 4.9 |
|  |  | Fmo5 | 4.1 | Gdf6 | 226.4 | Esr1 | 5.3 |
|  |  | Hspb1 | 4.8 |  |  | Tnfsf10 | 6.2 |
|  |  | Ctsb | 4.9 |  |  | Cxcr4 | 6.5 |
|  |  | Txnip | 5.1 |  |  | Atg9b | 10.0 |
|  |  | Fth1 | 5.4 |  |  | Tmem74 | 12.5 |
|  |  | Ucp2 | 5.5 |  |  | Ctss | 15.6 |
|  |  | Sod2 | 7.4 |  |  | Tgm2 | 21.4 |
|  |  | Sod3 | 9.6 |  |  |  |  |
|  |  | Cyp2f2 | 9.8 |  |  |  |  |
|  |  | Apoe | 11.0 |  |  |  |  |
|  |  | Ncf2 | 15.6 |  |  |  |  |
|  |  | Ccl5 | 21.4 |  |  |  |  |
|  |  | Xdh | 30.4 |  |  |  |  |
|  |  | Clu | 32.3 |  |  |  |  |
|  |  | Nos2 | 59.3 |  |  |  |  |
|  |  | Il19 | 256.8 |  |  |  |  |

**C**

| **OIM d21 vs WT d21** | | | | | | | |
| --- | --- | --- | --- | --- | --- | --- | --- |
| **osteogenesis** | | **Cell stress** | | **TGF-b** | | **autophagy** | |
| **gene name** | **fold change** | **gene name** | **fold change** | **gene name** | **fold change** | **gene name** | **fold change** |
| Sost | -50.8 | Hopx | -3.4 | Bglap2 | -26.7 | Dram1 | 2.9 |
| Bmp7 | -48.9 | Gsr | 2.4 | Dlx2 | -6.7 | Irgm1 | 4.0 |
| Gli1 | -37.8 | Fth1 | 2.6 | Ltbp4 | -5.9 | Tgm2 | 6.4 |
| Col10a1 | -21.8 | Sod2 | 5.9 | Gsc | -5.5 | Atg9b | 11.6 |
| Phex | -20.8 | Xdh | 9.7 | Gdf7 | -3.5 | Tmem74 | 39.2 |
| Gdf10 | -9.3 | Duox1 | 9.9 | Bambi | -2.9 |  |  |
| Col2a1 | -8.9 | Serpinb1b | 10.3 | Tgfb2 | -2.5 |  |  |
| Fgf1 | -5.9 | Ptgs2 | 12.4 | Tgfbr3 | 3.3 |  |  |
| Col1a2 | -5.6 | Ccl5 | 14.7 | Cdkn1a | 3.4 |  |  |
| Bmp3 | -5.0 | Il19 | 267.6 | Gdf6 | 37.5 |  |  |
| Itga2 | -4.1 |  |  | Il6 | 41.1 |  |  |
| Fgfr2 | -3.0 |  |  |  |  |  |  |
| Nog | -2.8 |  |  |  |  |  |  |
| Col4a1 | -2.7 |  |  |  |  |  |  |
| Tgfb2 | -2.5 |  |  |  |  |  |  |
| Cd36 | 2.2 |  |  |  |  |  |  |
| Vegfa | 3.7 |  |  |  |  |  |  |
| Csf2rb2 | 3.8 |  |  |  |  |  |  |
| Mmp8 | 6.7 |  |  |  |  |  |  |
| Tnfsf11 | 11.3 |  |  |  |  |  |  |
